# Fertility-Preserving Management of Uterine Arteriovenous Malformation in a 16-Year-Old Female

**DOI:** 10.7759/cureus.18162

**Published:** 2021-09-21

**Authors:** Harrison V Moynihan, Jordan Richardson, Kristian Loveridge

**Affiliations:** 1 Radiology, Detroit Medical Center, Detroit, USA; 2 Emergency Medicine, Detroit Medical Center Children’s Hospital of Michigan, Detroit, USA; 3 Interventional Radiology, Detroit Medical Center, Detroit, USA

**Keywords:** angiography, ultrasound, fertility preserving, arteriovenous malformation, embolization

## Abstract

Arteriovenous malformations (AVM) are abnormal connections between arteries and veins without a capillary bed, creating high- and low-flow areas that are prone to bleeding. Uterine AVMs can be congenital or acquired with an incidence of 0.1%. Acquired cases are usually caused by uterine instrumentation, trauma, infection, or gestational trophoblastic disease. Patients typically present with sudden onset of heavy vaginal bleeding. Diagnosis is made using angiography, ultrasound, computerized tomography, or magnetic resonance imaging. After patients are stabilized, management depends on their desire for future fertility and may include hysterectomy or endovascular embolization.

We present the case of a 16-year-old G1P0010 female with recurrent vaginal bleeding caused by a uterine AVM. To preserve the patient’s fertility, a selective embolization approach was employed using microcoils and gel foam. This case highlights a unique treatment option for uterine AVMs in patients who desire fertility preservation. Additionally, we review the diagnostic imaging and treatment options for uterine AVMs.

## Introduction

Abnormal uterine bleeding (AUB) is defined as menstrual bleeding that is abnormal and/or irregular in duration, frequency, or intensity [1]. AUB is a common chief complaint among patients presenting to emergency departments (ED). Although the etiology of AUB is broad, the International Federation of Gynecology and Obstetrics has developed a classification system to distinguish between structural and nonstructural causes. Structural causes include polyps, leiomyomas, adenomyosis, hyperplasia, and malignancy, while nonstructural causes include coagulopathy, ovulatory dysfunction, endometrial, or iatrogenic. Rarely, uterine arteriovenous malformation (AVM) is a cause of AUB.

Uterine AVMs can be a life-threatening cause of AUB [2]. Before the development of endovascular approaches to treat uterine AVMs, hysterectomy was the gold standard therapy to prevent life-threatening exsanguination [3]. For patients who desire fertility-preserving treatment, endovascular embolization by interventional radiology (IR) has become the mainstay of care for uterine AVMs [4-6].

Here, we present the case of a young 16-year-old G1P0010 female who presented to the ED several times for vaginal bleeding and symptomatic anemia. Pelvic magnetic resonance angiography (MRA) revealed a uterine AVM, which was confirmed by subsequent angiography carried out by IR. The patient desired fertility preservation and underwent successful definitive treatment of the uterine AVM using endovascular embolization.

## Case presentation

A 16-year-old G1P0010 female with recurrent vaginal bleeding presented to the ED with an episode of acute heavy vaginal bleeding. The patient reported soaking through multiple sets of clothes and passing golf ball-sized clots. The patient’s vital signs on presentation showed a blood pressure of 116/70 mmHg, pulse of 100 beats per minute, respiratory rate of 18 breaths per minute, and temperature of 37°C. The patient denied any episodes of lightheadedness, dizziness, or syncope. Pertinent laboratory values on presentation are illustrated in Table 1.

**Table 1:** Initial laboratory findings. RBC: red blood cell; MCV: mean cell volume; RDW: red cell distribution width; MCHC: mean corpuscular hemoglobin concentration; hCG: human chorionic gonadotropin

Lab marker	Finding
Hemoglobin	7.2 g/dL
Hematocrit	22.4 L/L
RBC	2.57 million/mm^3^
Platelets	249/µL
MCV	87.2 μm^3^
RDW	12.9%
MCHC	32.1 g/dL
hCG	6 mIU/mL

Physical examination revealed mild tenderness along the midline and both the right and left lower quadrants. On pelvic examination, there was active bleeding from the cervical os, which was closed. The patient experienced a significant drop in hemoglobin from her previous ED visit four days prior when her hemoglobin was 10.2 g/dL. This was her third visit to the ED in one month with similar complaints. The patient had a suction dilation and curettage (D&C) for voluntary termination of pregnancy two months prior and a repeat suction D&C one month prior for retained products of conception. Since discharge, the patient had reported continued mild-to-heavy vaginal bleeding with dark and bright red blood. At her last ED visit, she was admitted for evaluation. At that time, even though uterine AVM was discussed, repeat evaluation of the imaging was determined to be low risk and she was discharged home.

**Figure 1:**
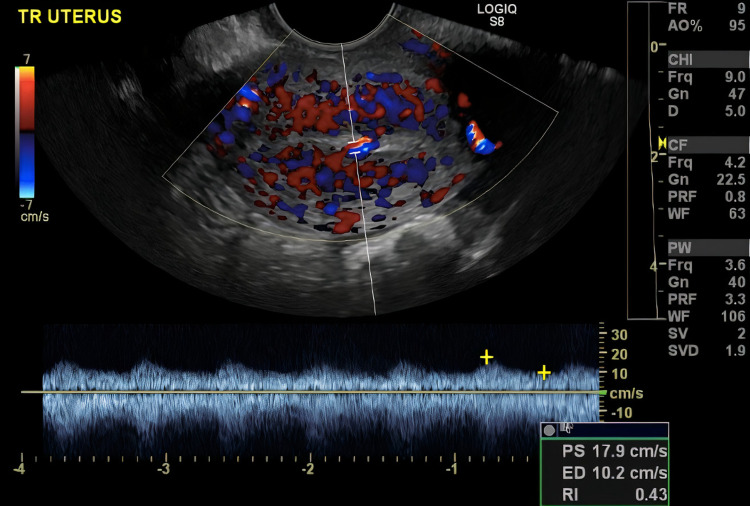
Transvaginal ultrasound with Doppler demonstrating uterine arteriovenous malformation.

Transvaginal ultrasonography (TVUS) was obtained which revealed a slightly thickened endometrium with increased vascularity and a hypoechoic, hypervascular focus in the endometrium. This led to a concern for retained products of conception or an AVM secondary to prior uterine surgeries (Figure 1).

**Figure 2:**
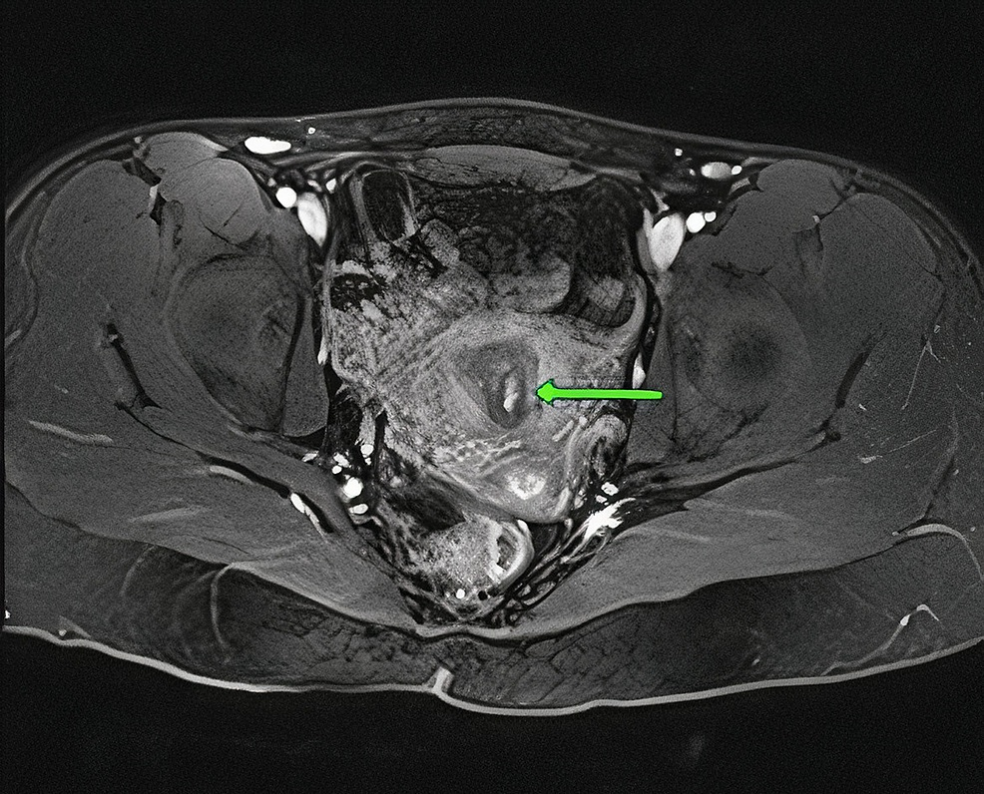
Pelvic MRI demonstrating arteriovenous malformation within the endometrial cavity.

**Figure 3:**
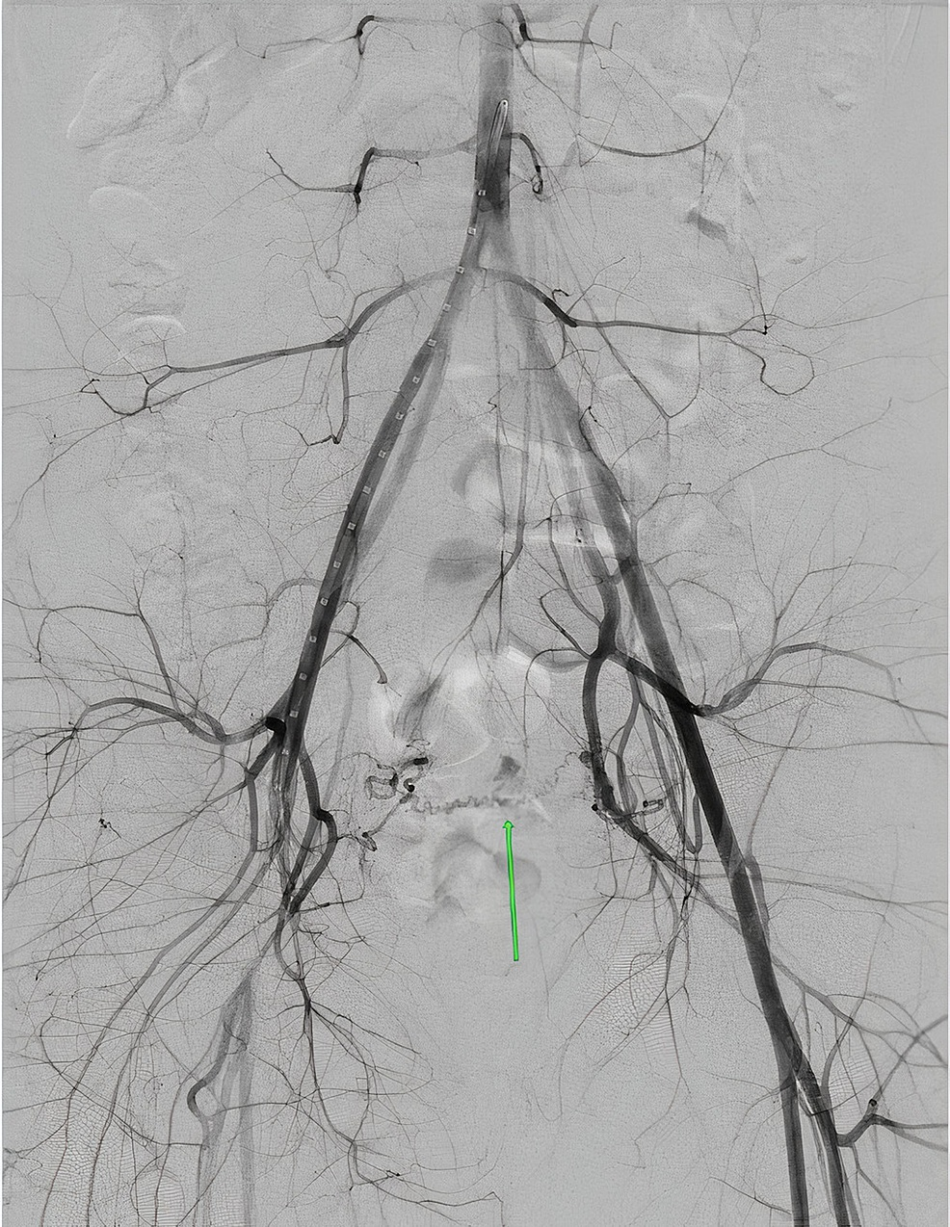
Pre-embolization pelvic angiogram demonstrating uterine arteriovenous malformation.

MRA was obtained and revealed abnormal vascularity suggestive of an AVM within the endometrial cavity at the junction between the body and the fundus of the uterus. Furthermore, a pelvic MRI was obtained which demonstrated an enhancing 8 × 4 mm polypoid structure within the endometrial cavity (Figure 2). These findings were suggestive of an AVM or post-traumatic changes from the patient’s prior uterine surgeries. IR was consulted for a diagnostic angiogram to confirm the suspicion of a uterine AVM. Prior to the patient’s diagnostic angiogram, she experienced an episode of vaginal bleeding with the passage of a medium-sized clot and her hemoglobin dropped to 5.9 g/dL. She was transfused with two units of packed red blood cells prior to the procedure. Transfemoral angiography was performed in the IR suite where pelvic angiography demonstrated an abnormal blush in the pelvis that corresponded with the location of the AVM (Figure 3).

**Figure 4:**
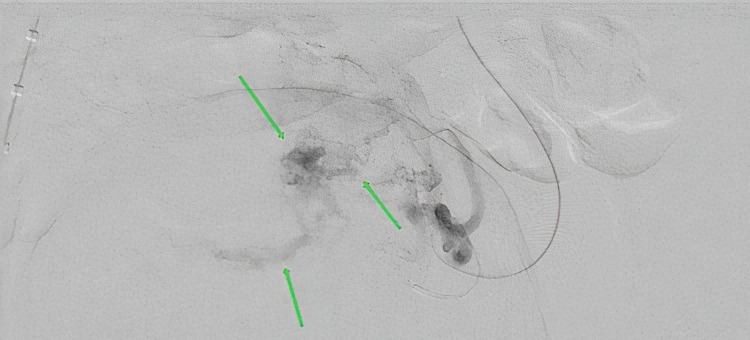
Angiography demonstrating malformation nidus, supplying artery, and draining vein of uterine arteriovenous malformation (green arrows from top to bottom, respectively).

Selective angiography of the left uterine artery confirmed an abnormal hypervascular nidus in the uterus which then gave rise to an early draining vein which drained into the right internal iliac vein (Figure 4).

Using the coaxial catheter technique, a microcatheter was advanced into the arcuate branch of the uterine artery supplying the AVM. Initial embolization was performed with a small volume of 900-1,200 µ Embosphere particles (Merit Medical, South Jordan, UT); however, given the brisk flow through the shunt, it was clear this would not be an optimal strategy. Therefore, n-butyl cyanoacrylate liquid embolic was also not used. The branch was embolized with detachable microcoils. Follow-up angiography confirmed occlusion with no further supply to the AVM via the left uterine artery.

**Figure 5:**
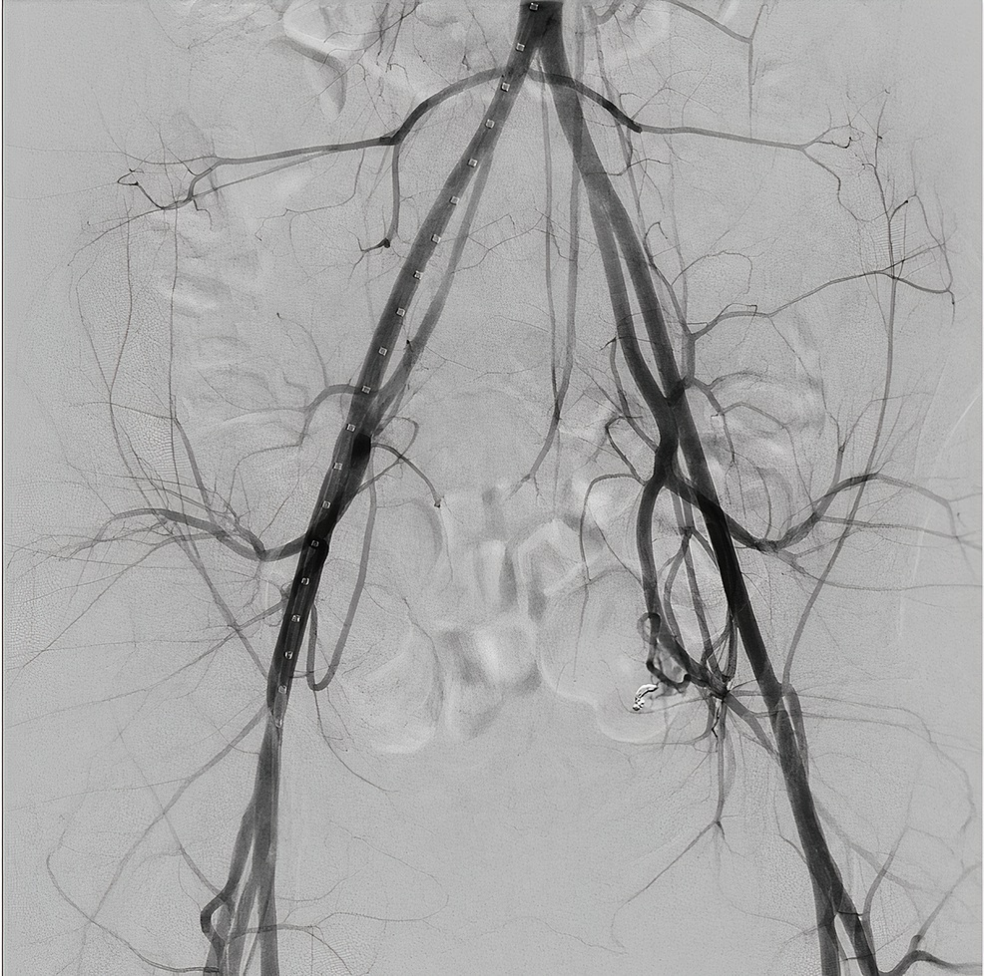
Post-embolization angiography demonstrating successful resolution of uterine arteriovenous malformation.

Angiography of the right internal iliac artery was performed, which showed persistent flow to the AVM from the right uterine artery. Selecting the right uterine artery was extremely difficult, and distal selection was not possible. Hence, embolization of the right uterine artery was performed with gel foam. The final pelvic angiography showed good perfusion of the uterine body and fundus as well as occlusion of the AVM (Figure 5).

The patient was re-evaluated the next morning after her procedure. She denied any vaginal bleeding or pain and did not report any post-procedure complications. Her hemoglobin was found to be stable at 9.3 g/dL upon discharge from the hospital.

## Discussion

We present the case of a young female patient with uterine AVM who was successfully treated with a fertility-preserving approach using embolization. A review of the literature revealed the average age of uterine AVM presentation to be approximately 30 years. Our patient was significantly younger, presenting at the age of 16. In young patients, the need for fertility-preserving treatment options is paramount.

Uterine AVMs can present with a plethora of symptomatology which poses a diagnostic challenge to clinicians. The most common presenting complaint is vaginal bleeding [3]. These patients generally have a history of uterine instrumentation. Abu Musa et al. reported a rare case of uterine AVM that presented with congestive heart failure secondary to a vascular steal syndrome [7]. At times, patients are completely asymptomatic. Fleming et al. reported a case of a uterine AVM that was found incidentally after hysterectomy [8]. Our patient had a classic presentation of uterine AVM with recurrent vaginal bleeding after two D&Cs for retained products of conception. Uterine AVM must be considered as a differential diagnosis in patients with AUB who do not respond to conventional management.

One of the first imaging modalities utilized in the management of AUB is TVUS. On TVUS, the uterine AVM demonstrates multiple irregular hypoechoic, tortuous, and tubular structures within the myometrium [9,10]. Ultrasound with color Doppler reveals a more specific image and shows a hypervascular area within the myometrium containing tortuous vessels with irregular turbulent flow [2,3]. Moreover, computed tomography with contrast and MRI have also been used for diagnosis [11,12]. However, the definitive diagnosis of uterine AVMs is made using angiography [3]. Angiography allows for direct visualization of any abnormal vascular formations, determines the feeding artery, and reveals early venous filling, which is a specific feature of uterine AVMs [13]. In our case, early venous drainage was appreciated on angiography, confirming the diagnosis of uterine AVM.

Retained products of conception and gestational trophoblastic disease are two important differentials that must be ruled out when AVM is suspected. Ultrasound can be used to delineate between these processes. Retained products of conception appear as a hyperechoic mass within the endometrial cavity and should be suspected when the endometrial wall thickness is more than 8-10 mm [9]. Moreover, beta-human chorionic gonadotropin levels are elevated in trophoblastic disease but normal in uterine AVM. Lastly, the presence of early venous draining on angiography is specific to AVM and is not appreciated in retained products of conception or gestational trophoblastic disease.

Acute treatment of uterine AVMs consists of controlling the hemorrhage. For patients who have uncontrolled, heavy uterine bleeding, a Foley bulb can be inserted into the uterus to tamponade the bleeding [14]. Brown et al. suggested that intravenous estrogen may help control the bleeding [15]. The definitive treatment for uterine AVM depends on the patient’s plans for future fertility. Until 1982, hysterectomy was the gold standard treatment. Advances in IR have been responsible for the increased prevalence of fertility-preserving endovascular treatment options. Selective embolization of uterine arteries is considered the first-line fertility-preserving treatment in the management of symptomatic uterine AVMs with success rates of up to 94.1% [16,17]. Medical management is also an alternative option for uterine AVMs. Methylergonovine maleate, tranexamic acid, combined oral contraceptive pills, danazol, and tropin-releasing hormone agonists are promising alternatives to surgical management [14]. In our case, the resolution of vaginal bleeding and uterine AVM was achieved by selective embolization of bilateral uterine arteries. During a two-month follow-up outpatient visit, no recurrence of the AVM was discovered and the patient remained symptom-free.

Menstrual cycles are expected to reappear within one to two months after selective embolization of uterine AVMs [16]. Regarding fertility preservation, there are several reports of successful intrauterine pregnancies following uterine artery embolization; however, there is a higher risk of intrauterine growth restriction and prematurity [16-18].

## Conclusions

Uterine AVM is a rare cause of AUB and should always be considered on the differential diagnoses in women with a history of uterine instrumentation due to its potential life-threatening nature. TVUS with color-flow Doppler utilization is an effective, noninvasive, first-line detection modality among women. Endovascular selective embolization of uterine arteries has proven to be a safe and effective treatment option for symptomatic uterine AVMs. Successful utilization of this treatment modality may prolong or circumvent entirely the need for a hysterectomy. Especially in patients as young as described in this case, this fertility-preserving procedure has profound ramifications when patients with AVM are of child-bearing age and should be considered as the first-line option.
